# 3D-printed implantable devices with biodegradable rate-controlling membrane for sustained delivery of hydrophobic drugs

**DOI:** 10.1080/10717544.2022.2057620

**Published:** 2022-04-01

**Authors:** Camila J. Picco, Juan Domínguez-Robles, Emilia Utomo, Alejandro J. Paredes, Fabiana Volpe-Zanutto, Dessislava Malinova, Ryan F. Donnelly, Eneko Larrañeta

**Affiliations:** a School of Pharmacy, Queen’s University Belfast, Belfast, UK; b Wellcome-Wolfson Institute for Experimental Medicine, Queen's University Belfast, Belfast, UK

**Keywords:** Olanzapine, implant, 3D-printing, sustained delivery

## Abstract

Implantable drug delivery systems offer an alternative for the treatments of long-term conditions (i.e. schizophrenia, HIV, or Parkinson’s disease among many others). The objective of the present work was to formulate implantable devices loaded with the model hydrophobic drug olanzapine (OLZ) using robocasting 3D-printing combined with a pre-formed rate controlling membrane. OLZ was selected as a model molecule due to its hydrophobic nature and because is a good example of a molecule used to treat a chronic condition schizophrenia. The resulting implants consisted of a poly(ethylene oxide) (PEO) implant coated with a poly(caprolactone) (PCL)-based membrane. The implants were loaded with 50 and 80% (w/w) of OLZ. They were prepared using an extrusion-based 3D-printer from aqueous pastes containing 36–38% (w/w) of water. The printing process was carried out at room temperature. The resulting implants were characterized by using infrared spectroscopy, scanning electron microscopy, thermal analysis, and X-ray diffraction. Crystals of OLZ were present in the implant after the printing process. *In vitro* release studies showed that implants containing 50% and 80% (w/w) of OLZ were capable of providing drug release for up to 190 days. On the other hand, implants containing 80% (w/w) of OLZ presented a slower release kinetics. After 190 days, total drug release was ca. 77% and ca. 64% for implants containing 50% and 80% (w/w) of OLZ, respectively. The higher PEO content within implants containing 50% (w/w) of OLZ allows a faster release as this polymer acts as a co-solvent of the drug.

## 1. Introduction

Implantable drug delivery systems provide long-acting drug release, offering higher bioavailabilities than conventional routes of administration (Langer, 1990; Dash & Cudworth, 1998; Rajgor et al., 2011). Therefore, this type of drug delivery system has great potential due to their ability to reduce potential side effects (Zhou et al., 2015; Y. Wang et al., 2020). Furthermore, these systems improve the efficacy and tolerability of treatment, providing a better quality of life to patients. This is especially important for patient’s suffering for chronic conditions. Chronic conditions are medical conditions that last at least more than 12 months limiting patient’s life activities and requiring constant medical attention. There are a wide variety of chronic conditions such as schizophrenia, HIV (Gendelman et al., 2019; Li et al., 2021), or Parkinson’s disease (Govender et al., 2017). The treatment of these conditions normally requires constant drug uptake. As a result of the continuous pharmacological treatment, a relatively high percentage of cases present treatment adherence problems. Factors associated with poor adherence include higher relapse rates, increased hospitalization, poorer quality of life and higher levels of residual symptoms (Parellada & Bioque, 2016). Non-adherence to medications also has an economic impact (Hong et al., 2011). It has been previously demonstrated that non-adherence to treatment has a large economic impact in terms of hospitalization expenses or treatment (Hong et al., 2011; Van Boven et al., 2014; Ho et al., 2016; Lin et al., 2021). Accordingly, implantable drug delivery systems can be used to provide sustained drug release for patients who need treatments for period of time ranging from weeks to years (Delivery, 2012).

The classification of implantable devices can be complex due to the large variety of devices and geometries described in the literature. A conventional way of classifying implantable devices separates them into two categories: matrix-type and reservoir-type implants. The difference between these systems is based on their structure. In the matrix form, the drug is homogeneously dispersed through the polymer. On the other hand, in reservoir-type implants the drug is located within the core of the implant and a membrane controls the release of the drug (A. S. Stewart et al., 2018; S. Stewart et al., 2020). However, it is important to mention that there are exceptions to this classification method as some implants present the drug distributed within different parts of the implant (Zhao et al., 2022).

Reservoir-type implants are likely to provide sustained release without showing an initial burst drug release. However, present several disadvantages. These rate controlling membranes are commonly produced using non-biodegradable polymers. Accordingly, once the drug is fully released or when treatment discontinuation is required they have to be removed (Rabin et al., 2008; Schlesinger et al., 2016). The use of biodegradable implants could prevent the need for implant removal improving patient wellbeing while reducing healthcare costs.

Implantable devices can be prepared using a wide variety of techniques, including hot-melt extrusion, injection molding, and 3D-printing (A. S. Stewart et al., 2018; Domsta & Seidlitz, 2021; Z. Wang & Yang, 2021). The use of 3D-printing technologies has been widely explored for the manufacture of a wide range of drug delivery systems such as implantable devices, oral dosage forms, or suppositories (Mathew et al., 2019; Awad et al., 2020; Domínguez-Robles et al., 2020; Melocchi et al., 2020; S. Stewart et al., 2020; S. A. Stewart et al., 2020; Borandeh et al., 2021). Moreover, this family of technologies has the potential to produce structures of precise shapes from a 3D model by deposition of material in a layer-by-layer fashion, thus providing the ability to manufacture patient specific implantable devices (Chen et al., 2017; Martin et al., 2021). The high degree of flexibility and controllability of this approach could be used to produce a tailored and accurate treatment regime designed to exactly match the individual patient and condition to be treated (Khaled et al., 2014; S. Stewart et al., 2020; S. A. Stewart et al., 2020; Domínguez-Robles et al., 2021).

In this work, we propose the combination of 3D-printed implants and biodegradable rate controlling membranes to produce devices capable of providing sustained drug delivery. The implantable drug delivery system developed in this study was composed of a thin poly(caprolactone) (PCL) film coating and a poly(ethylene oxide) (PEO) core containing olanzapine (OLZ) as a drug model. A robocasting 3D-printer was used to prepare the OLZ/PEO-based implants. The implants were extensively characterized, and drug release was evaluated for periods of 6 months.

## 2. Materials and methods

### 2.1. Materials

OLZ powder was provided by Cangzhou Enke Pharma-Tech Co. Ltd. (Cangzhou, China). PEO 100,000, dichloromethane, and acetonitrile were obtained from Sigma-Aldrich (Dorset, UK). PCL Capa^TM^ 2054 (MW = 550 g/mol) and PCL Capa^TM^ 6506 (MW = 50,000 g/mol) were provided by Ingevity (North Charleston, SC). Acetic acid was obtained from Honeywell (Charlotte, NC) while sodium azide was obtained from Fluorochem Ltd. (Hadfield, UK).

### 2.2. Preparation of implantable devices

OLZ/PEO implants were prepared by dissolving PEO in water using a SpeedMixer™ DAC 150.1 FVZ-K (Hauschild GmbH & Co. KG, Westfalen, Germany) at 3000 rpm for two minutes. Then, different amounts of OLZ were added and mixed again with the previous mixture for another two minutes at 3000 rpm. The water content of the formulation was 36–38% (w/w) while two different OLZ/PEO ratios were prepared: 50/50 (50% OLZ/PEO) and 80/20 (80% OLZ/PEO). Then, the mixtures were transferred into a 10 mL syringe equipped with an 18 G plastic tip and placed in the extruder of Allevi^®^ 3D-printer. Samples were printed at room temperature using the following parameters: 2 mm/s of speed, 0.6 mm of thickness layer, and 38 PSI of pressure. Implant design was prepared using CAD-based software. Implants containing no drug, only PEO, were prepared using the same methodology. As an alternative, a low temperature 3D-printing method was developed using a Bioscaffolder 3.2 (GeSiM, Radeberg, Germany) robocasting equipment. For this purpose, solid mixtures of OLZ/PEO (50/50) were loaded into a piston-based extruder equipped with a 0.5 mm nozzle. Two different printing temperatures were used 60 and 80 °C. The layer height was set at 0.25 mm and the print speed was set at 10 mm/s.

PEO-based implants were wrapped with a PCL membrane. For this purpose, a membrane containing a mixture of two PCL with different molecular weights was prepared as described previously (S. A. Stewart et al., 2020). The selected mixture contained 40% of higher molecular weight PCL (PCL 6506) and 60% of lower molecular weight PCL (PCL 2054). The mixture of PCLs was dissolved in dichloromethane (7% w/w) and then it was deposited in a petri dish until all the solvent was evaporated to obtain films. Film thickness was 0.16 ± 0.02 mm. Finally, implants were fabricated by wrapping the implant with the PCL films and sealing the ends with a hot gripper.

### 2.3. Characterization of implants

#### 2.3.1. Microscopic examination

The implantable devices were viewed using a Leica E24W digital microscope (Leica, Wetzlar, Germany). Moreover, a tabletop scanning electron microscopy (SEM) (Hitachi TM3030, Tokyo, Japan) was used to evaluate the morphology of the implants and films. The SEM analysis was carried out in low vacuum mode at a voltage of 15 kV and without sample pretreatment.

#### 2.3.2. Differential scanning calorimetry and thermogravimetric analysis

The implants and pure OLZ and PEO were analyzed using differential scanning calorimetry (DSC). DSC was performed using a Q100 differential scanning calorimeter (TA Instruments, Bellingham, WA). Scans were run from 25 °C to 225 °C with a heating rate of 10 °C/min under a nitrogen flow rate of 50 mL/min. Moreover, a Q500 Thermogravimetric analysis (TA Instruments, Bellingham, WA) was used to characterize the samples. For this purpose, samples were between 25 °C and 500 °C with a heating rate of 10 °C/min under a nitrogen flow rate of 40 mL/min.

#### 2.3.3. Attenuated total reflectance Fourier transform-infrared spectroscopic analysis (ATR-FTIR)

A Spectrum Two FT-IR Spectrometer (Perkin Elmer, Waltham, MA) was used along with MIRacle™ Single Reflection attenuated total reflectance (ATR) (Pike Technologies, Fitchburg, MA) with a MIRacle™ Confined Space Clamp to analyze the implants and the powders. Spectra were recorded from 4000 to 600 cm^−1^ with a resolution of 4 cm^−1^. All the spectra presented in this work were obtained as an average of 32 scans.

#### 2.3.4. X-ray diffraction assay

The crystallinity of the OLZ powder and implants was assessed using a Miniflex™ X-ray powder diffractometer (Rigaku Corporation, Tokyo, Japan) equipped with Ni-filtered, Cu Kβ radiation, at a current of 15 mA and a voltage of 30 kV as described previously (Volpe-Zanutto et al., 2021).

#### 2.3.5. Optical coherence tomography

The implant devices were assessed by optical coherence tomography (OCT). To this purpose, the implant covered with the film was placed on a plate and analyzed using an EX1301 OCT microscope (Michelson Diagnostics Ltd., Kent, UK).

### 2.4. Drug release experiment

The release study was performed for 190 days. Implants were placed in vials containing 50 mL of PBS (pH: 7.4) at 37 °C and agitated at 40 rpm. The experiment was carried out under sink conditions. Sodium azide was used to prevent the growth of microorganisms in the media. At defined time points, the release media was replaced with fresh one and then, the quantity of OLZ in the media was analyzed using reverse-phase high-performance liquid chromatography (RP-HPLC). For this purpose, an Agilent 1100 series system HPLC (Agilent Technologies UK Ltd., Stockport, UK) equipped with a Waters X-Select CSH C18 column (3.5 µm pore size, 3.0 × 150 mm) (Agilent Technologies UK Ltd., Stockport, UK) was used to quantify OLZ. The mobile phase consisted of a mixture of acetonitrile and water (pH 2.3) at a ratio of 60:40. The flow rate was 5 mL/min, injection volume of 10 µL, sample runtime of 5 min and UV detection was at 260 nm.

### 2.5. Cytocompatibility of PCL-based membranes

HEK293T (human embryonic kidney, ATCC) cells were seeded in DMEM (Dulbecco’s modified Eagle medium), supplemented with 10% fetal calf serum and non-essential amino acids, in 24-well plate at 30,000 cells per well. Following overnight incubation, an equal volume of culture media pre-incubated with a piece of the PCL-film. On day 3 post treatment, cells were imaged on Olympus widefield microscope using ×20 objective. Moreover, on the same day, cell viability was measured using MTT assay (3-(4,5-dimethylthiazol-2-yl)-2,5-diphenyltetrazolium). Briefly, wells were washed in PBS and MTT solution added to each well and incubated for three hours. Viable cells convert water soluble MTT to insoluble formazan, producing colored precipitate (Serrano et al., 2004; Elamparithi et al., 2016). Absorbance at 570 nm was measured using a plate reader (Biotek, Winooski, VT). In addition to this, MTT assay was performed for samples on day 7 post treatment.

### 2.6. Statistical analysis

All measurements were expressed as a mean ± standard deviation. Statistical analysis was carried out using *t*-test. A statistical level of *p*<.05 was considered statistically significant.

## 3. Results and discussion

### 3.1. Preparation and characterization of OLZ-loaded implantable devices

**Figure 1.**
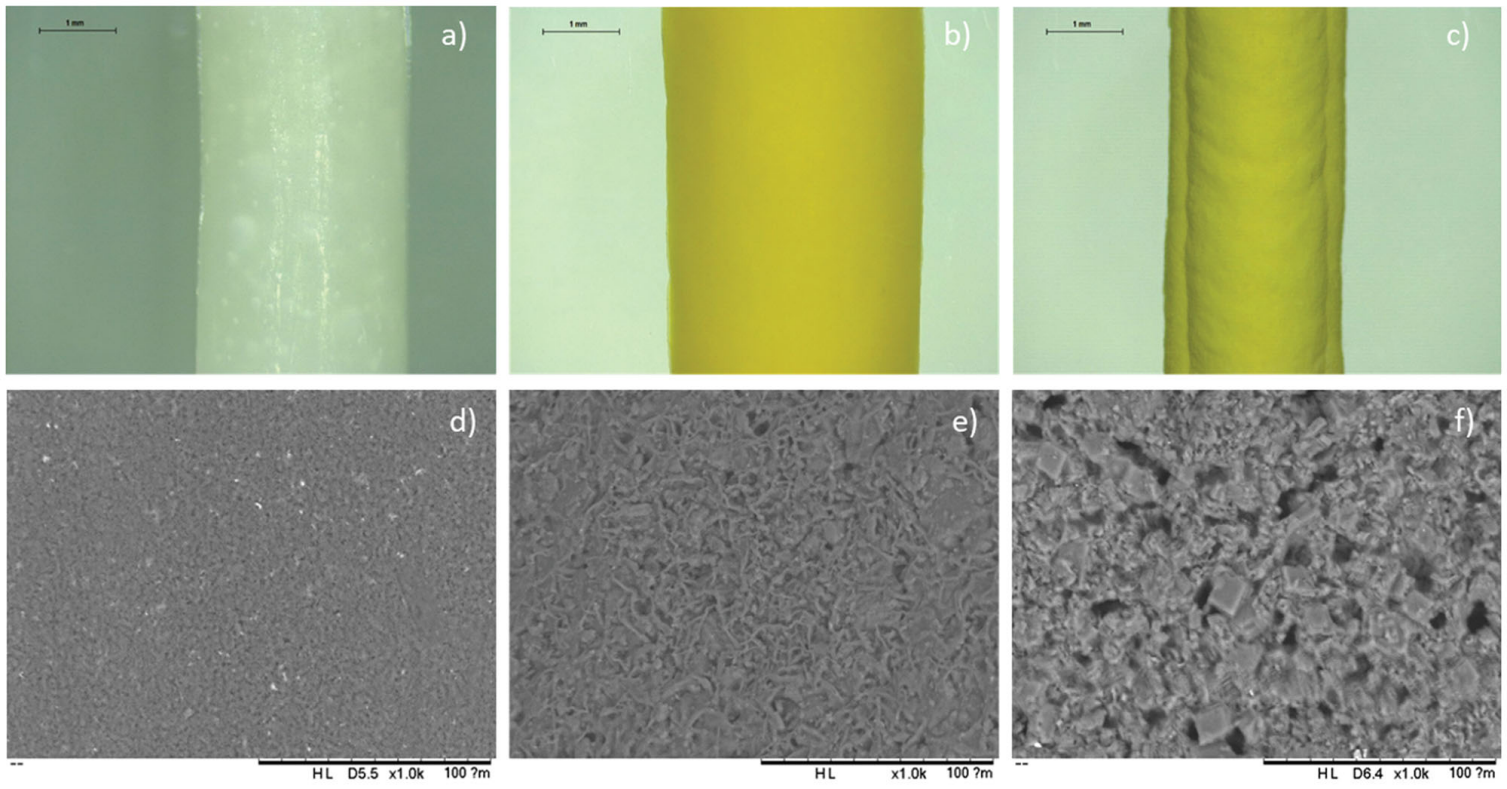
Images of implants using the Leica E24W microscope at ×16 magnification of (a) PEO implant, (b) 50% OLZ/PEO implant, and (c) 80% OLZ/PEO implant. Scale bar: 1 mm. SEM images of (c) PEO implant, (d) 50% OLZ/PEO implant, and (e) 80% OLZ/PEO implant at ×1000 magnification with a scale of 100 µm.

Implants containing 50 and 80% (w/w) of OLZ as a model drug were prepared using an extrusion-based robocasting 3D-printing technology. The formulation was a viscous paste containing PEO and OLZ in water. Subsequently, water was allowed to evaporate to obtain a solid device. This type of approach was described previously for the manufacturing of solid oral dosage forms (Khaled, Alexander, Wildman, et al., 2018). The composition of the formulation used for the printing process is important to ensure the correct manufacturing of the implants. The addition of higher amounts of OLZ yielded formulations with higher viscosity. Accordingly, the resulting implants had a better-defined geometry (thinner implants) as the mixture did not flow as much when extruded. On the other hand, the formulation containing 50% (w/w) of the drug presented lower viscosity. Therefore, the resulting implants were broader (Figure 1).

#### 3.1.1. Microscopy study

Figure 1 shows the morphology of the implants prepared using different OLZ loadings. It can be observed that OLZ was well dispersed throughout the entire implant. The resulting implants showed a yellow color due to the presence of the drug. The yellow color of the implants containing 80% (w/w) of OLZ was darker, confirming that a higher amount of OLZ is present in the prepared implants. Thus, it can be inferred that the drug was homogeneously dispersed throughout the PEO matrix.

OLZ orodispersible films have been prepared before using 3D-printing technology (Cho et al., 2020). This type of formulation contained lower drug loadings (5% w/w) and were prepared using a hot-melt extrusion technique requiring high temperatures ranging between 160 and 170 °C. The approach proposed in this work requires can be used to print implants at room temperature. This is an ideal approach for thermolabile drugs or for peptides/proteins. In parallel, we evaluated the use of a hot-melt extrusion 3D-printing method to prepare implants with high OLZ content at 60 and 80 °C. The printing process could not be completed at 60 °C, as the samples could not be extruded. Interestingly, the material was extruded from the barrel despite of the large drug content at 80 °C. However, the 3D-printing process could not be completed as the layers of extruded material did not adhere to form the 3D-object. Accordingly, we believe that the method proposed here is more suitable for the preparation of dosage forms with high drug content.

SEM was used to evaluate the surface morphology of the resulting implants (Figure 1(d–f)). The images showed the presence of drug crystals on the surface of the implants, which were also well dispersed within their structure, as no obvious drug crystal aggregates were observed. Moreover, blank implants showed the presence of PEO crystals that have a smaller size than OLZ crystals (Figure 1(f)). Shape and size of OLZ crystals can provide information about the crystalline form of the drug. Crystals with plate-like square shape suggest the presence of crystalline form I (Luo et al., 2019). However, crystalline form needs to be confirmed using information from other techniques such as XRD. The presence of OLZ crystals within the surface of implants has been reported before. Recently, de Almeida et al. (2021) reported a hot-melt extrusion method for the production of PCL/OLZ implants. These implants contained lower drug loading than the one reported here (23% w/w), and drug crystals were found in the surface of the implants. Alternatively, implants made of drug crystals dispersed within a polymeric material have been previously reported for different drugs including levothyroxine and islatravir (also known as MK-8591) (Barrett et al., 2018; S. A. Stewart et al., 2021).

#### 3.1.2. Differential scanning calorimetry and thermogravimetric analysis (DSC-TGA)

**Figure 2.**
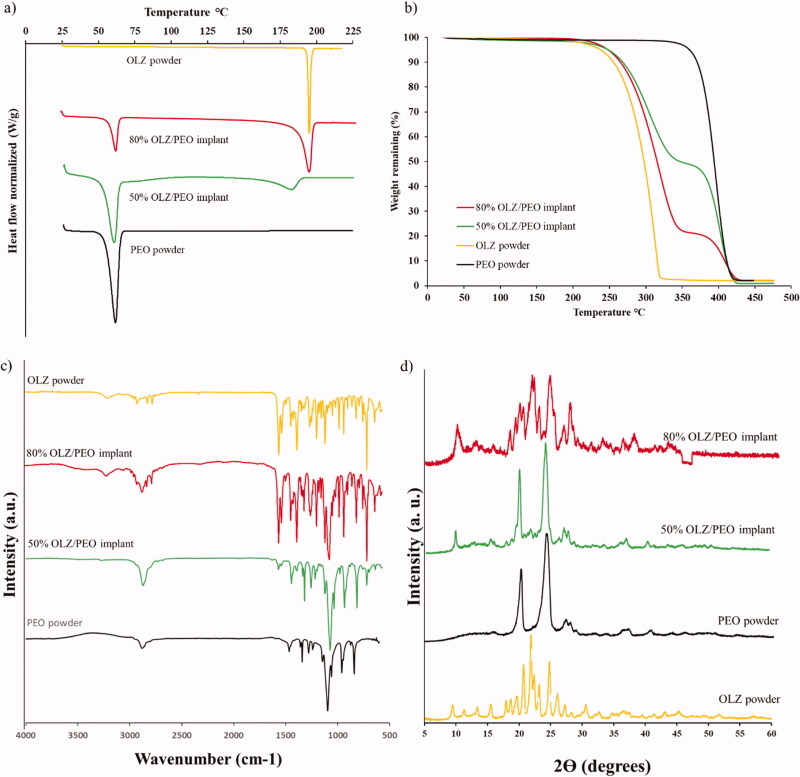
(a) DSC curve of OLZ powder, 80% OLZ/PEO implant, 50% OLZ/PEO implant and PEO powder, (b) TGA curve of PEO powder, 50% OLZ/PEO implant, 80% OLZ/PEO implant and OLZ powder. (c) IR spectra of OLZ powder, 80% OLZ/PEO implant, 50% OLZ/PEO implant, and PEO powder, showing % transmittance over wavenumber range 4000–600 cm^−1^. (d) X-ray spectra of implants with different percentages of the drug, OLZ powder and PEO powder.

Information about the crystalline properties of OLZ powder, PEO powder, and all implants was obtained by thermal analysis. The DSC curves show an endothermic melting peak for both PEO and OLZ at 60 °C and 195 °C, respectively (Figure 2(a)). These results indicate that the raw materials used to prepare the implants presented crystalline domains. Alternatively, DSC curves for the implants containing OLZ and PEO show equivalent endothermic peaks but with lower melting temperatures and intensities. These results are indicating that there are some crystallinity changes and potential interactions may have occurred between the drug and the polymer. Finally, the implants showed a broader melting peak for OLZ, and they have shifted toward lower temperatures, which could be attributed to a thermally induced amorphization of the blend during DSC experiment (Paredes et al., 2020).

TGA curves (Figure 2(b)) revealed that the *T*_onset_ of pristine PEO (above 350 °C) was higher than the *T*_onset_ for OLZ and samples containing 50 and 80% (w/w) of OLZ (above 200 °C). Although, 3D-printed implants contained 20 and 50% (w/w) of PEO, the *T*_onset_ of these samples was similar to the one obtained for OLZ. Moreover, these 3D-printed samples showed two clear degradations stages, unlike PEO and OLZ. The first degradation (233.92 °C) can be attributed to the degradation of OLZ while the second one (358.14 °C) can be attributed to PEO degradation. It is important to note that the weight loss after OLZ degradation is consistent with drug content. These results indicate not only drug stability during the printing process, but that water was properly evaporated. Moreover, this can be confirmed by the absence of weight loss between 25 and 100 °C (Paredes et al., 2021). Therefore, these results are indicating that, although some interactions may have occurred, 3D-printed samples are forming a dispersion of OLZ in the PEO matrix. However, the presence of a melting peak for implants containing OLZ confirms the presence of OLZ crystals in the implants, as observed in SEM images (Figure 1(d–f)).

#### 3.1.3. Attenuated total reflectance Fourier transform-infrared spectroscopic analysis

FTIR analysis was performed to evaluate any potential interactions between OLZ and PEO within the 3D-printed implants (Figure 2(c)). The spectrum of pristine PEO exhibited characteristic peaks at 3160 cm^−1^, 2905 cm^−1^, and 1125 cm^−1^ that can be assigned to the C–H bonds, CH_2_ bonds and a triplet (C–O–C), respectively, as has been previously reported (Sim et al., 2010). Moreover, the FTIR spectrum of pure OLZ revealed characteristic peaks at 3200 cm^−1^, 1601 cm^−1^, and 754 cm^−1^ that can be assigned to N–H bonds (Testa et al., 2019), C=N bonds (Ayala et al., 2006), and C–H bonds (Laing et al., 2020), respectively. OLZ peaks can be found not only in the IR spectrum of 3D-printed implants containing 80% (w/w) of OLZ, but also in the 3D-printed samples containing 50% (w/w) of OLZ. In addition, the latter also showed the characteristic peaks found in the spectrum of pristine PEO. No new peaks were obtained suggesting that no chemical reaction took place during the mixing or the extrusion-based 3D-printing process.

#### 3.1.4. X-ray diffraction assay

XRD patterns of OLZ powder, pristine PEO and 3D-printed implants containing both drug loadings (50 and 80%) are shown in Figure 2(d). Pure OLZ showed numerous sharp narrow peaks that indicate high crystallinity. Some of the characteristic peaks of OLZ could be seen at 9.48, 21.94, and 26.14 2*Ɵ* degrees but with different diffraction intensities. The existence of these peaks in the implants samples means that OLZ has been well incorporated and its crystalline nature was still maintained. Characteristics peaks of PEO appears in the implant loaded with 50% (w/w) of OLZ at 20.22 and 24.12° 2*Ɵ* degrees approximately with high intensity, whereas, in the implant, with 80% (w/w) of OLZ these peaks have lower intensity due to the lower amount of the polymer. As mentioned previously, the SEM crystal shapes suggested the presence of the OLZ polymorph I. XRD results confirmed this as pure drug presented characteristic peaks of this polymorph at 8.6°, 12.4°, 14.4°, and 16.9° (Testa et al., 2019). Interestingly, the crystalline form is maintained after the printing process as some of these peaks can be found in the samples containing 50 and 80% (w/w) of OLZ. Solid dosage forms prepared via-3D-printing using pastes presented similar behavior (Khaled et al., 2015; Khaled, Alexander, Irvine, et al., 2018). On the other hand, hot-melt extrusion based approaches for the production of OLZ/loaded orodispersible films or implants yielded amorphous drug dispersions (Cho et al., 2020). However, drug content was lower than that described in the present work (5 and 23% w/w).

#### 3.1.5. Implant assembly

**Figure 3.**
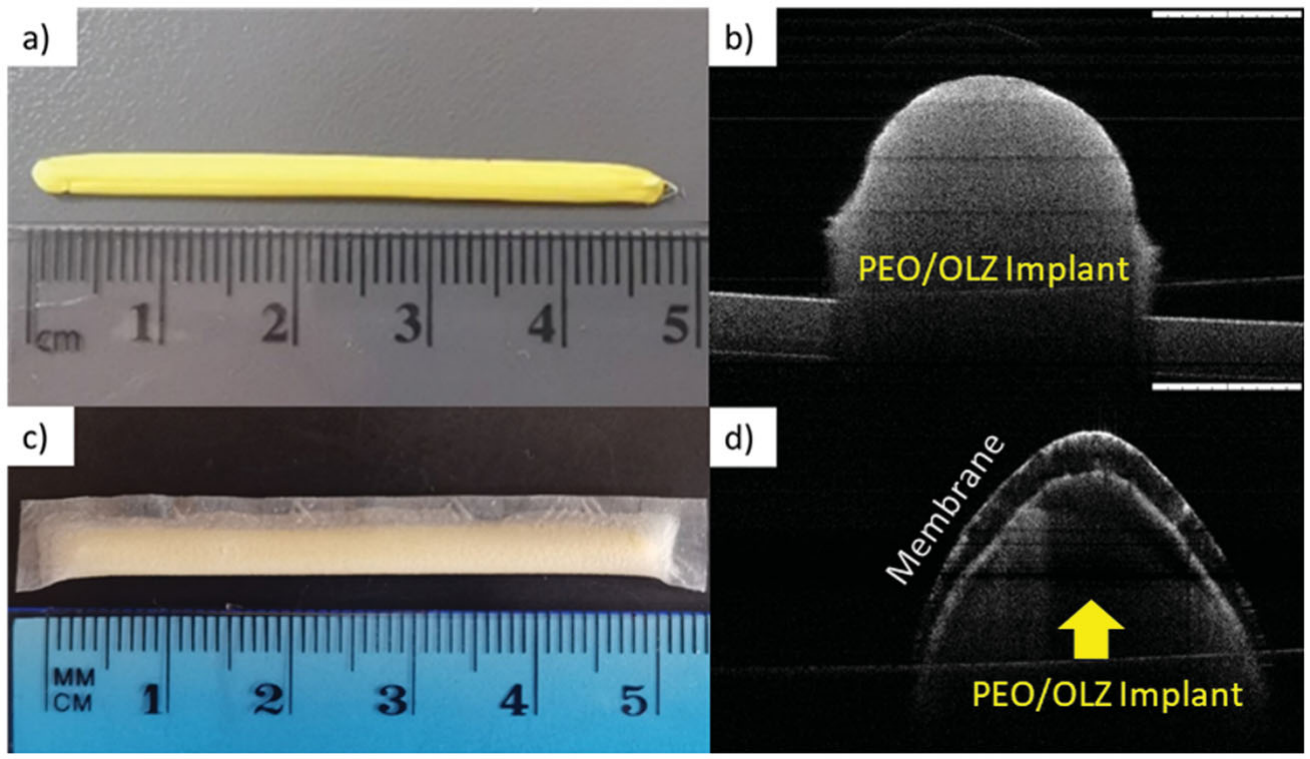
(a) Picture of OLZ/PEO implant without the film, (b) OCT of OLZ/PEO implant without the film, (c) image of final device, and (d) optical coherence tomography (OCT) of OLZ/PEO implant with the film. Scale bar OCT images: 1 mm.

OCT is a technique normally used to evaluate biological tissue such as the eye or the skin. However, the applications of this technique for medical device characterization have been extensively described. In this case, the laser provides information about the different parts of the implant. Figure 3(a,b) shows the implant without the film coating, in which it could be seen that the implant is solid, uniform and without gaps inside. The outer sections of the implants are brighter than the inner ones due to the laser penetration. However, no pores or holes were detected. On the other hand, Figure 3(c,d) shows the final release system. In this case, an external layer corresponding to the film with a solid mass corresponding to the implant is observed. The inner part of the device containing the PEO-based implant is not as bright as in Figure 3(b) due to light penetration across the membrane. This image indicates that the film was able to adapt to the morphology of the implant and suitably coat it. This particular type of film was selected based on previous work developed for preparing implants for sustained release of hydrophilic molecules (S. A. Stewart et al., 2020). As the use of this type of membrane for release of hydrophobic compounds remains unexplored, it was selected to control the release of OLZ from 3D-printed implants.

This type of devices can be implanted in the upper arm in a similar way to contraceptive implants. Other potential application sites are the dorsal area or the abdomen (Chua et al., 2018; Delpor, 2022). A good example of this are titanium implants developed by Delpor^®^ (Pons-Faudoa et al., 2019). The implants developed by this company present similar sizes (4 cm × 4 mm) than the one described here (Delpor, 2022). These implants are currently been studied for the delivery of hydrophobic drugs (Delpor, 2020). Accordingly, the implants developed in this work presented sizes that are in line with previously developed implantable devices.

### 3.2. Release study

**Figure 4.**
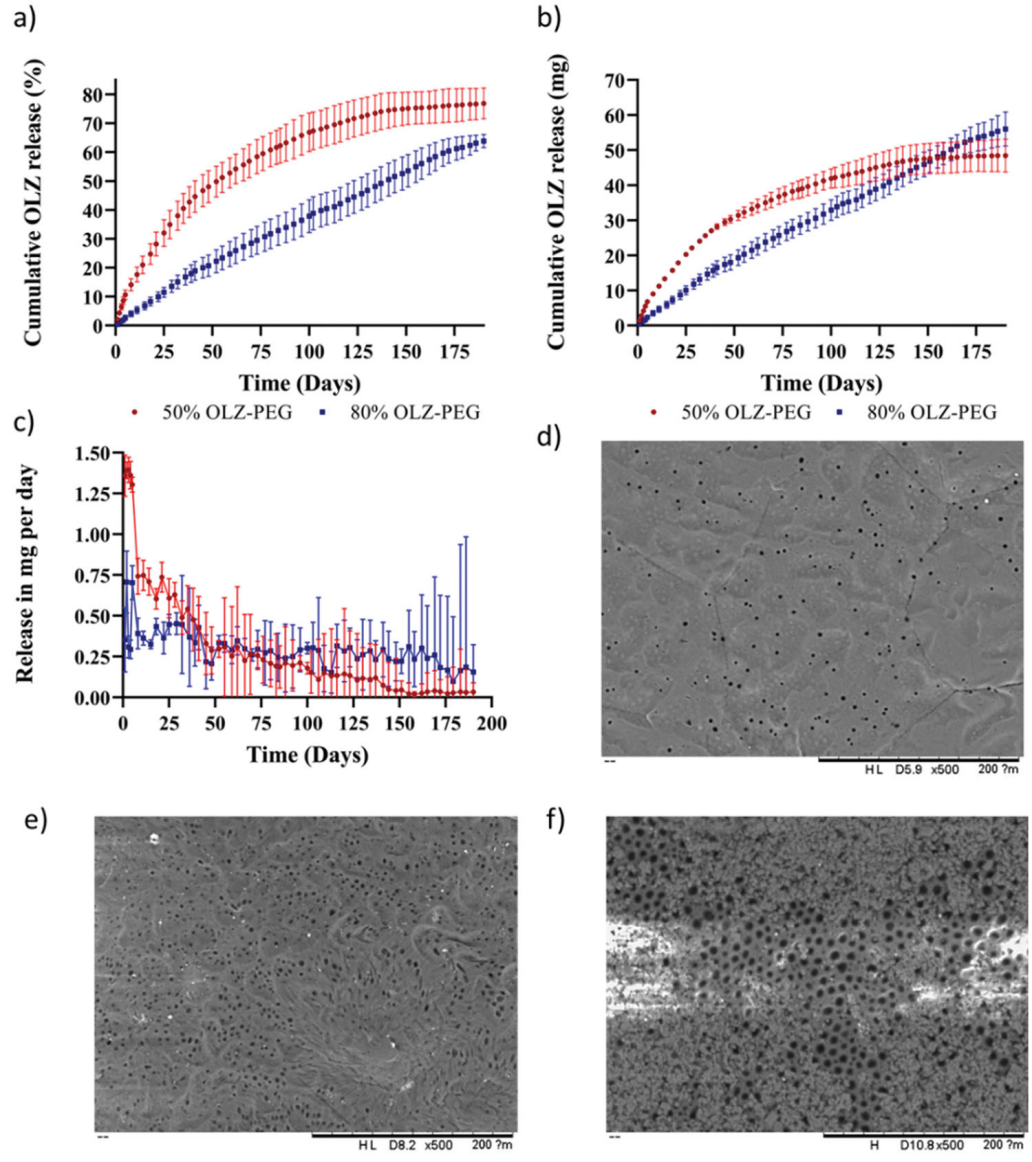
Graphic of (a) cumulative release implants at 50 and 80% (w/w) of OLZ in percentage, (b) cumulative release implants at 50 and 80% (w/w) of OLZ in milligrams, and (c) release in mg per day implants at 50 and 80% (w/w) of OLZ. SEM images of film at 500 magnifications, before (d) and after 190 days, (e) for the film coating implants loaded with 50% (w/w) of OLZ, and (f) for the film that coated implants loaded with 80% (w/w) of OLZ. The scale in this images is 200 µm.

*In vitro* release was studied for implants containing 50 and 80% (w/w) of OLZ wrapped with the PCL-based film. OLZ release from the 3D-printed implants was evaluated for 190 days (Figure 4). Both formulations showed a sustained drug release for 190 days and no obvious burst release was observed within the first days of release. This factor is important considering that OLZ/PEO implants disintegrate when placed in PBS solution after PEO dissolution. This experiment was performed with OLZ/PEO materials. After 24 h, the amount of OLZ dissolved was 67 ± 4% for implants containing 50% (w/w) OLZ while for implants containing 80% (w/w) OLZ the percentage of drug dissolved was 58 ± 8%. These values are lower than the ones obtained for the PCL coated implants that were lower than 2.5% in both cases. Accordingly, the PCL membrane is required to obtain sustained drug release.

When observing the release curves, implants containing 50% (w/w) of OLZ showed a faster release within the first 40–50 days. After this period of time, the release rate was reduced until the system reached a plateau after 190 days. However, these two different regions were not observed in the release profile of the implant formulation containing 80% (w/w) of OLZ. These implants showed a linear drug release for 190 days. Figure 4(c) shows the daily release rate of the implants. These results suggest that implants containing 80% (w/w) of OLZ present a constant drug release rate over prolonged periods of time. On the other hand, implants containing 50% (w/w) of OLZ presented a higher drug release rate during the first 50 days and then a gradual decline. The release rate at day 50 was equivalent for both types of implants (*p*> .05) while the release rate at days 100 and 150 was higher for implants containing 80% (w/w) of OLZ. Finally, the release rate decline for both types of implants showing equivalent release rate at day 190 (*p*> .05). The amount of OLZ released and the percentage of drug released for these two types of implants were compared at different time points: 50, 100, 150, and 190 days. The cumulative amount of OLZ released during at the first 100 days was higher for implants containing 50% (w/w) of OLZ. This was evidenced after comparing the amount of OLZ released at day 50 and day 100 (*p*< .05). Interestingly, the total amount of OLZ released after 150 days and 190 days was equivalent (*p*> .05). When comparing the release of OLZ comparing the percentage of drug released both types of implants showed statistically significant differences when comparing the release after 50, 100, 150, and 190 days (*p*< .05).

These implants consisted of a dispersion of OLZ in a PEO-based polymeric matrix coated by a rate controlling membrane. The release process requires several steps. First, water has to permeate trough the membrane. Subsequently, water inside the implant should dissolve the drug. Finally, dissolved drug should diffuse out. Implants containing 50% (w/w) of OLZ showed a faster release because they contained higher amount of PEO that can contribute to OLZ solubilization as has been previously described (Cho et al., 2020). On the other hand, implants containing 80% (w/w) of OLZ presented lower PEO content and therefore the total concentration of OLZ dissolved inside the implant was lower. Therefore, drug concentration gradient played a key role in drug release. These results are consistent with previously reported implants containing PLC-based rate controlling membranes that showed linear prolonged drug release for more than 200 days for different compounds such as tenofovir alafenamide or model molecules such as methylene blue (Li et al., 2020; S. A. Stewart et al., 2020).

OLZ has been used as a model hydrophobic compound for the development of implantable drug delivery systems. Although OLZ is mainly used for the treatment of psychoses and related disorders, such as schizophrenia (National Health Service (NHS), 2021), low-dose OLZ can be a promising treatment for other medical conditions such as cancer-related anorexia (Okamoto et al., 2019), anorexia nervosa (Dunican & DelDotto, 2007; Leggero et al., 2010), or acne excoriée (Gupta & Gupta, 2001). Moreover, drug dose can be increased by adding more than one implant formulation, as has been previously reported (Karunakaran et al., 2021). The use of multiple implants is a possibility but is not ideal. In order to minimize the application, impact the number of implants should be kept as low as possible. For this purpose, changing OLZ for another drug requiring lower dosage can be beneficial. A few examples of low dose drugs used to treat chronic conditions are risperidone (schizophrenia, 1–3 mg/day), levothyroxine (hypothyroidism, 100 µg/day), or rivastigmine (Alzheimer's disease, 1–2 mg/day)

The morphology of the films before and after the 190-day release experiment was observed using the electronic microscope and the results are presented in Figure 4(d–f). The original film presented pores in its structure that were homogeneously distributed across the surface of the implant. After the release process, the number of pores increased as can be seen in Figure 4(b,c). This is consistent with previously published work that described the use of this type of films for hydrophilic drug release (S. A. Stewart et al., 2020). Interestingly, the films that were used to coat 80% (w/w) of OLZ implants presented a larger pore size distribution. The porosity of the films was attributed to the hydrolysis of the ester bond of the PCL (Lam et al., 2009). The weak base nature of OLZ produces an increment in the pH of the micro-environment of the film which leads to a greater extent of degradation of ester bonds of PCL (Rydholm et al., 2007; Sailema-Palate et al., 2016). As described previously 50% (w/w) of OLZ implants presented higher drug release rates during the first 50–75 days of release due to the effect of a higher PEO content. However, for implants containing 80% (w/w) of OLZ, the membrane was exposed to an aqueous environment with high OLZ concentrations for longer periods of time. Accordingly, we believe that this phenomenon explains the higher degree of porosity of the membranes. It is important to note that the degradation of this type of PCL membranes will not compromise the stability of the implant for periods up to 300 days due to a slow degradation kinetic (S. Stewart et al., 2020; S. A. Stewart et al., 2020).

### 3.3. Cytocompatibility study of PCL-based membranes

The polymers used to prepare the implants and the membranes are FDA approved polymers so no cytocompatibility issues were expected. PEO is an FDA approved pharmaceutical excipient (Gref et al., 1995). Additionally, OLZ depot forming injections have been approved by the FDA (Lindenmayer, 2010). However, PCL membranes were prepared using dichloromethane as a solvent. High concentrations of this solvent within a medical/pharmaceutical product can lead to toxicity issues. Accordingly, it is crucial to evaluate the biocompatibility of the final material. Despite of potential toxicity issues with this type of solvent, it has been used for tablet coating and its use is tolerated by the U.S. FDA (Sohi et al., 2004; FDA, 2017). However, solvent residual levels should be measured before the product can be marketed (FDA, 2017).

**Figure 5.**
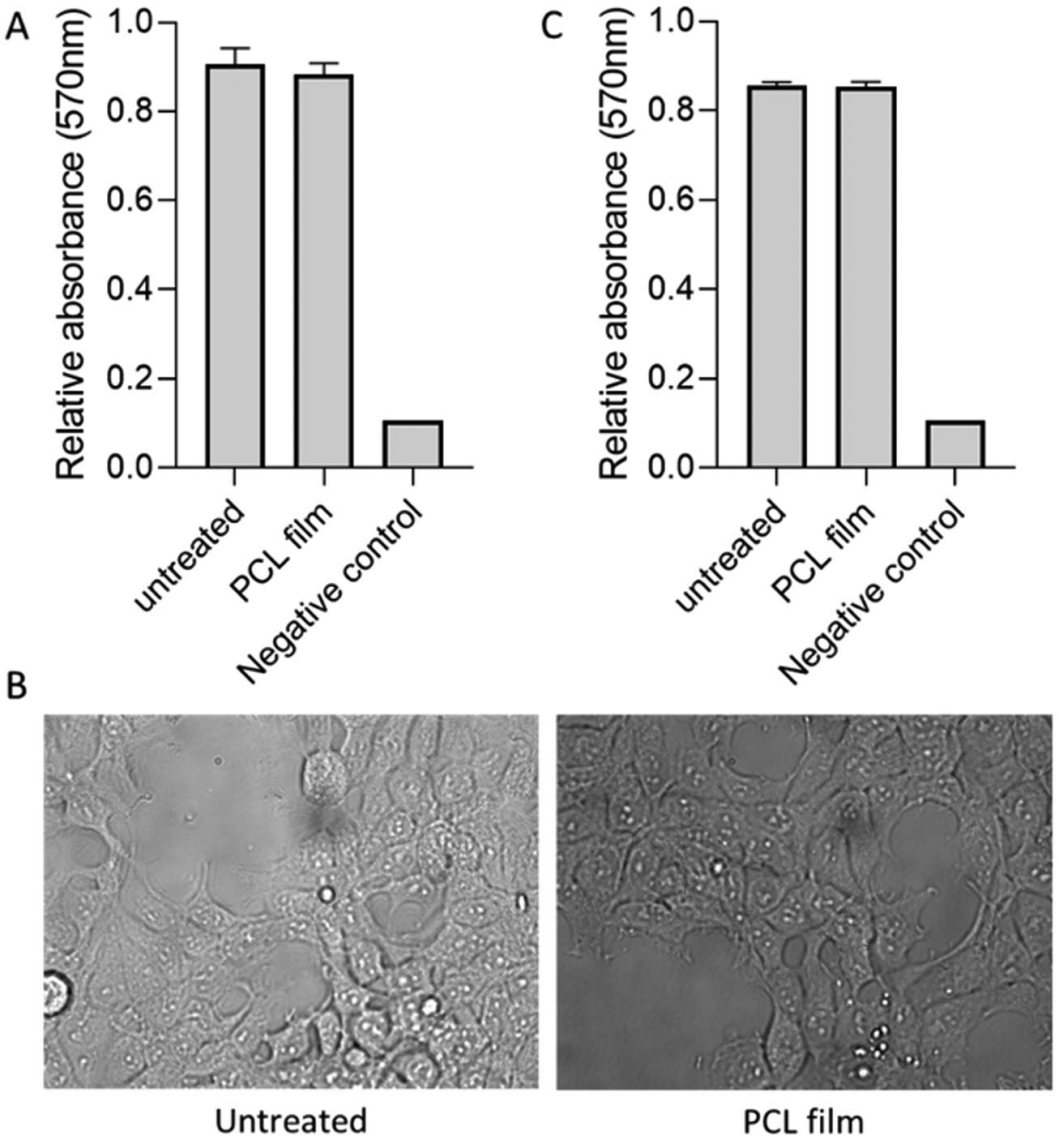
Cytocompatibility test for PCL-based membranes on HEK293T cells. MTT assay on day 3 post treatment (A). Microscopy images of cells on day 3 after for untreated and cells treated with PCL film (B). MTT assay on day 7 post treatment (C).

In order to evaluate potential toxicity issues associated with the solvent HEK293T cell line was used. This cell line has been previously used to treat cytocompatibility of different types of medical devices and pharmaceutical formulations (Avti et al., 2013; Burugapalli et al., 2016). Cytocompatibility results can be seen in Figure 5. Results confirm no effect of treatment on viability of human adherent HEK293T cells three days post treatment (*p*> .05). Moreover, images were obtained after three days showing normal cell adhesion, spreading and morphology in HEK293T cells exposed to PCL film. Size cell, granularity, and coverage of the well surface were indistinguishable from control untreated cells. Finally, the MTT assay was repeated at longer times (seven days post treatment) showing no significant differences between the untreated group and the PCL film group. These results suggest that the resulting implants are cytocompatible. These results are promising but more work is needed to ascertain the safety of these devices including animal experiments.

These membranes are safe despite of requiring dichloromethane to be prepared. A potential solution to avoid the use of this solvent will be to prepare this type of devices by using solvent-free technologies. An alternative could be the use of injection molding PCL-based prepared rate-controlling tubular implants that can be subsequently combined with the PEO-implant. However, the structure of the resulting membranes needs to be tested as these membranes could potentially present differences to the ones described in this work.

## 4. Conclusions

In this work, a combination of 3D-printing method and biodegradable rate controlling membranes has been described to prepare implants for sustained drug delivery. Implantable drug delivery systems of OLZ were successfully produced using extrusion-based 3D-printing technology. Two formulations containing 50 and 80% (w/w) of OLZ were prepared. The 3D-printed implants were subsequently coated with a biodegradable polymeric film to act as a rate controlling membrane. This film was made from a mixture of two types of PCL polymers. Implants containing 50% (w/w) of OLZ and thus the highest amount of PEO showed a faster release than the formulation containing 80% of OLZ and 20% of PEO during the first 75 days. This could be explained by the effect of PEO as a co-solvent for the drug. Therefore, the solubility of OLZ increased as the amount of PEO increased. Moreover, implants containing 80% (w/w) of OLZ showed a linear drug release with no burst release after 190 days. The results presented in this work demonstrate the potential of this implantable drug delivery systems for sustaining the release of hydrophobic compounds, such as OLZ. This drug is not only used for the treatment of psychoses and related disorders, such as schizophrenia, but also could be a promising treatment for other medical conditions, such as cancer-related anorexia, anorexia nervosa, or acne excoriée. The methodology described here to prepare implants is simple and does not require using high temperatures unlike hot-melt extrusion or injection molding. More work is required before this technology can be clinically applied. The first step would be its evaluation in an animal model. Additional work is required to evaluate drug release of other compounds from this type of implant. Ideally, drugs with lower dose requirements for the treatment of chronic conditions could be ideal candidates.

Despite these promising results, there are still unanswered questions regarding the use of 3D-printing for the development of medical devices such as drug eluting implants. Important aspects such as implant sterilization or even quality control/reproducibility need to be addressed before 3D-printing technology can be applied as a point-of-care technology. Nevertheless, due to the potential of this type of technology the FDA is working actively to provide guidance to researchers and companies.
